# Interaction between type 2 diabetes and past COVID-19 on active tuberculosis

**DOI:** 10.21203/rs.3.rs-3989104/v1

**Published:** 2024-03-14

**Authors:** Liz E. Calles-Cabanillas, Genesis P. Aguillón-Durán, Doris Ayala, José A. Caso, Miguel Garza, Mateo Joya-Ayala, America M. Cruz-Gonzalez, Raul Loera-Salazar, Ericka Prieto-Martinez, Javier E. Rodríguez-Herrera, Esperanza M. Garcia-Oropesa, John M. Thomas, Miryoung Lee, Jordi B. Torrelles, Blanca I. Restrepo

**Affiliations:** University of Texas Health Science Center at Houston; University of Texas Health Science Center at Houston; University of Texas Health Science Center at Houston; University of Texas Health Science Center at Houston; University of Texas Health Science Center at Houston; University of Texas Rio Grande Valley; Secretaria de Salud de Tamaulipas; Secretaria de Salud de Tamaulipas; Secretaria de Salud de Tamaulipas; Secretaria de Salud de Tamaulipas; Universidad Autónoma de Tamaulipas; University of Texas Rio Grande Valley; University of Texas Health Science Center at Houston; Texas Biomedical Research Institute; University of Texas Health Science Center at Houston

**Keywords:** tuberculosis, COVID-19, Type 2 diabetes, Mexico, cross-sectional study

## Abstract

**BACKGROUND:**

The global setback in tuberculosis (TB) prevalence and mortality in the post-COVID-19 era have been partially attributed to pandemic-related disruptions in healthcare systems. The additional biological contribution of COVID-19 to TB is less clear. The goal of this study was to determine if there is an association between COVID-19 in the past 18 months and a new TB episode, and the role played by type 2 diabetes mellitus (DM) comorbidity in this relationship.

**METHODS:**

A cross-sectional study was conducted among 112 new active TB patients and 373 non-TB controls, identified between June 2020 and November 2021 in communities along the Mexican border with Texas. Past COVID-19 was based on self-report or positive serology. Bivariable/multivariable analysis were used to evaluate the odds of new TB in hosts with past COVID-19 and/or DM status.

**RESULTS:**

The odds of new TB were higher among past COVID-19 cases vs. controls, but only significant among DM patients (aOR 2.3). The odds of TB given DM was 2.7-fold among participants without past COVID-19 and increased to 7.9-fold among those with past COVID-19.

**CONCLUSION:**

DM interacts with past COVID-19 synergistically to magnify the risk of TB. Latent TB screening and prophylactic treatment, if positive, is recommended in this COVID-19/DM/latent TB high-risk group.

## INTRODUCTION

Tuberculosis (TB), a lung infection caused by *Mycobacterium tuberculosis (Mtb),* is a healthcare challenge in low- and middle-income countries. The World Health Organization (WHO) has reported a global setback in efforts to control TB following the coronavirus disease 2019 (COVID-19) pandemic [1]. This includes a fall in the number of reported TB cases during 2020, followed by increasing rebounds in 2021 by 28%, and in 2022 by 16%, when it reached levels higher than any year prior to 2019 [1]. During this 2020 to 2022 period, COVID-19 related disruptions caused an excess of half a million deaths due to TB [1]. These excess TB cases and deaths are attributed in part to disruptions in national healthcare systems [2]. At the biological level there are also potential biological interactions between COVID-19 and host comorbidities such as type 2 diabetes mellitus (DM), that can increase TB risk and complicate prognosis [3–6]. Thus, it is important to understand the COVID-related factors reversing TB control efforts at an individual level.

The population along the United States (US)-Mexico border has more TB when compared to the 2019 national incidences per 100,000 in the US (9.3 vs 2.7) and Mexico (35.0 vs 17.7) [7–10]. Factors driving vulnerability to develop TB in these border regions include migration, poverty, limited access to healthcare and weakened immune system due to DM and obesity [11–13].

A weakened immune system may occur after a COVID-19 episode due to persistent inflammation, cytokine dysregulation, and lymphopenia that can make the host permissive to secondary infections [14–16]. Individuals with underlying medical conditions such as DM, have an increased risk of COVID-19 development, severity, and mortality [17]. Further social disparities that favor TB risk at the Mexico border also contribute to the deleterious impact of COVID-19 pandemic [18].

Given the high vulnerability of the US-Mexico border population for lung infections, this setting is uniquely posited to test the hypothesis that individuals with a recent COVID-19 episode would have a higher risk of developing TB. We conducted a cross-sectional study among newly diagnosed TB patients and non-TB controls to determine whether a recent COVID-19 episode would be more likely to occur in TB. Our findings show that the odds of TB are higher in participants with past COVID-19, but results are only significant in individuals with DM. We discuss the clinical implications of these findings for mitigating the odds of TB in new COVID-19 patients.

## METHODS

### Study design and participant characterization

A cross-sectional study was conducted during the first 18 months of the COVID-19 pandemic (June 2020 to November 2021) in the Mexican cities of Reynosa and Matamoros, across the border with Texas, United States (US). Participants were HIV-negative adults (age ≥ 18 years). Pulmonary TB patients and their close contacts were identified at pulmonary clinics from the Secretaría de Salud Tamaulipas (SSA), and additional non-TB controls were recruited from the same Hispanic communities.

Newly diagnosed pulmonary TB was based on a positive sputum smear for acid-fast bacilli, culture for *Mtb*, or clinical diagnosis (abnormal chest x-rays and symptoms). Close contacts shared at least 5 hours of airspace with a new TB patient but no evidence of TB. Community controls did not have a history of TB nor known exposure to a TB case in the past 2 years. Evidence of latent TB infection in non-TB controls was based on a positive Interferon Gamma Release Assays [**IGRA**; QuantiFERON-Gold in-Tube (Qiagen) or T.Spot-TB (Oxford Immunotec)]. BCG vaccination was based on scarring.

A past history of COVID-19 (current cases not included) was based on self-report due to symptoms suggestive of COVID-19 during the past 18 months and/or a positive serology for anti-SARS-CoV-2 nucleocapsid IgG index (hereafter ‘COVID-IgG’; Alinity c test platform, Abbott Laboratories). COVID-19 vaccination became available during the study period and participants were considered vaccinated if they received at least one dose.

Participant sociodemographics, physical measures, self-reported medical history and laboratory testing were documented as described [19]. DM was based on self-report, hyperglycemia or chronic hyperglycemia (HbA1c ≥ 6.5%) [20]. Macrovascular (heart diseases, high blood pressure) and microvascular diseases (neuropathy, kidney disease) were self-reported. Body mass index (BMI) was stratified into underweight or normal (< 24.9 kg/m^2^) and overweight or obese (> 25 kg/m^2^).

### Statistical Analysis

Pearson’s chi-square was used to determine associations between categorical variables and Fisher’s Exact was used when any cell sample size was ≤ 5. The t-test was used to analyze differences in means for continuous variables. For multivariable models, variables with p ≤ 0.2 by univariable analysis or of biological interest (e.g. sex, age, BMI) were entered into backwards regression models to identify the predictors of TB in addition to past COVID-19. DM was of particular interest and was evaluated as a confounder or through a multiplicative effect modifier assessment. Variables with p ≤ 0.05 were kept in the final reduced model. All statistical analysis was performed using Statistical Analysis System (SAS) version 9.4. P values ≤ 0.05 were considered significant and between 0.05 and 0.09 borderline significant.

## RESULTS

### Definition of past COVID-19

**Figure 1:**
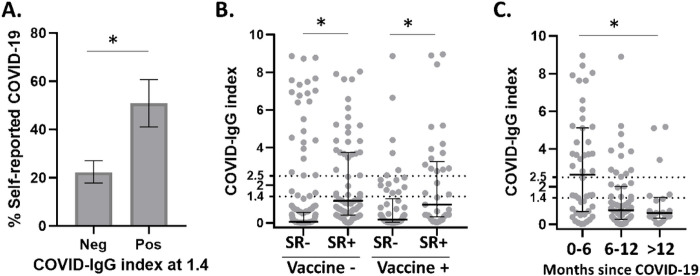
Anti-SARS-CoV-2 serology (COVID-IgG index) in all participants with respect to COVID-19 report, vaccination and episode timing. **A**. Prevalence of self-reported COVID-19 among individuals with a negative or positive COVID-IgG index cut-off of 1.4 per manufacturer recommendations. Error bars, 95% CI. **B**. COVID-IgGindex among study participants by COVID-19 self-report (SR + or −) or vaccination status. Dotted horizontal lines show cut-offs of 1.4 (manufacturer recommendation) and 2.5 (this study). **C**. COVID-IgG index stratified by the time elapsed between enrollment and months elapsed since the reported COVID-19 episode. *, p ≤ 0.05. **SR**: self-reported COVID-19; **COVID-Ig index**: anti-SARS-CoV-2-IgG index; **Neg**:: Negative; **Pos**: Positive.

We studied 485 participants (112 TB cases, 284 close contacts, 89 community controls). The first step was to define past COVID-19 based on self-reported disease (n = 478, 98.6% interviewed) and COVID-IgG (n = 445, 91.8% tested; **Table S1**). When using the COVID-IgG cut-off of 1.4 per manufacturer recommendations, self-reported COVID-19 was more prevalent among individuals with a positive *vs*. a negative serology (50.9% *vs*. 22.2%; p < 0.001; Fig. 1A). Likewise, positive COVID-IgG was more likely among those reporting a COVID-19 history vs. no history (OR 3.64, 95% CI: 2.31–5.75; Table 1). However, 53 individuals had positive serology but no reported COVID-19, suggesting some COVID-19 cases were asymptomatic.

During this study period the COVID-19 vaccines became available (n = 370, 76.3%; **Table S1**), so vaccination history was taken into consideration for defining COVID-19 given its possible effect on serology or mitigation of COVID-19 symptoms. COVID-19 vaccinees had higher COVID-IgG (Fig. 1B), and particularly among those who did not report COVID-19 (positive COVID-IgG using the clinical cut-off of ≥ 1.4: 24.2% in vaccinees vs. 16.1% in non-vaccinees; Table 1). These associations were not significant but suggested a partial influence of the COVID-19 vaccine on positive serology. To increase the specificity of the serology, we evaluated a higher IgG index cut-off. We selected 2.5 based on visual inspection of the titers in individuals with self-reported COVID-19 and vaccination history (Fig. 1B). This higher IgG index cut-off provided a more specific estimate of infection: a shift from 24.2% to only 9.8% of vaccinees having a positive serology among those with no self-reported COVID-19 (OR 0.64; 95% CI: 0.25–1.66; Table 1).

We noted that IgG titers were highest in individuals with recent COVID-19 episodes and waned over time [IgG index median, IQR at 0–6 months = 2.64 (4.40); 6–12 months = 0.75 (1.72), and 12 or more months = 0.60 (1.01); Fig. 1C)]. Accordingly, the proportion of individuals with a positive serology also decreased over time among individuals with reported COVID-19 (0–6 months: 55.6%; 6–12 months: 35.2%; ≥ 12 months: 9.26%; p = 0.004). These findings suggested that as longer times elapsed since a COVID-19 episode, false-negative serology was more likely.

Together, our observations suggested a partial overlap between a positive COVID-19 history and serology, and an influence of vaccination history and timing since COVID-19 on serology titers. Hence, our final classification of subjects with past COVID-19 was based on reported history of disease in the last 18 months or a COVID-IgG cut-off ≥ 2.5. Hereafter, we refer to this new variable of symptomatic and asymptomatic cases as “past COVID-19”.

### Characteristics of participants by TB status

For analysis by TB status, we combined the 284 close contacts and 89 community controls into one non-TB control group given the small size of the latter group. TB cases vs. non-TB controls had a higher proportion of males (TB 72.3% vs no TB 35.7%; p < 0.001), lower education (TB 25.9% vs. non-TB 39.0% with ≥ high school degree; p < 0.011) and less smoking (TB 6.3% vs. non-TB 17.4%; p < 0.004). For health-related conditions, TB cases had more DM (TB 56.3% vs. non-TB 23.9%; p < 0.001), and microvascular complications (TB 34.5% vs. non-TB 19.3%; p < 0.001) and yet, lower BMI (underweight/normal BMI: TB 75.9% vs. non-TB 22.3%; p < 0.001). Laboratory analyses indicated that TB patients vs non-TB controls had lower lipids, hemoglobin, hematocrit, and lymphocyte counts, while higher monocytes, platelets, and neutrophils (p < 0.001)(**Table S2**).

### Association between past COVID-19 and pulmonary TB

The goal of this study was to determine if past COVID-19 was associated with TB, but we found no significant differences with any COVID-19 related variable, i.e. reported COVID-19, COVID-IgG, vaccination, or the composite past COVID-19 variable (**Table S2**)). However, upon further stratification of participants by past COVID-19 and TB, the features that distinguished past COVID-19 patients were higher odds of marriage among non-TB, or higher DM or high HbA1c among TB patients (**Table S3**). Blood white blood cell counts did not differ by past COVID-19 and TB status (**Table S4**)), but among non-vaccinated TB contacts, lymphocyte counts were lower in those with past COVID-19 vs a negative COVID-19 history (**Fig S1**).

### Role of DM on the association between past COVID-19 and TB

Further analysis was conducted to evaluate the relationship between TB, DM and past COVID-19. Bivariable analysis suggested that past COVID-19 was an effect modifier of the association between TB and DM (Table 2). Namely, the odds of TB was 4.10 among DM compared to no DM in all participants, 2.71 among those negative for past COVID-19 episode, and 7.85 among those with a past COVID-19 episode. Table 3 shows the independent contribution of past COVID-19 to TB in multivariable models. Model A included all the variables and model B was the reduced version. Both models indicated that past COVID-19 was not significantly associated with TB in the overall sample. However, Model C showed a borderline interaction between past COVID-19 * DM (p 0.057). Similar models among participants with DM (Model D) or poor glucose control (HbA1c ≥ 7.0% in model E and ≥ 7.5% in model F) showed that the strength of the association between past COVID-19 and TB increased among participants with poor glucose control (DM adjusted OR, aOR 2.33; HbA1c ≥ 7.0 aOR 2.99; HbA1c ≥ 7.5 aOR 3.31). Together, these findings suggest that past COVID-19 alone is not independently associated with TB, but this association is significant among DM patients, and particularly in those with poor glucose control.

## Discussion

**Figure 2:**
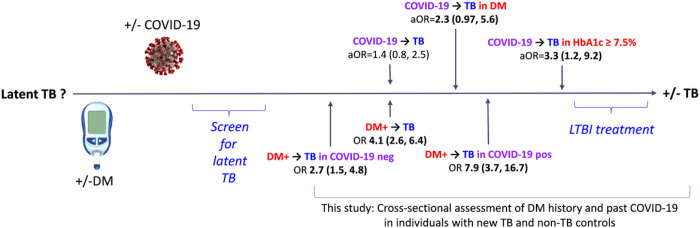
Summary of findings on the interaction between COVID-19 and DM on the odds of TB. Among all participants the odds of TB were not significantly higher in individuals with past COVID-19, but increased among those with DM (aOR 2.3), and become significant in those with poor glucose control (aOR 3.3). Additionally, the odds of TB was nearly 3-fold in DM vs. non-DM patients without past COVID-19 and increased to nearly 8-fold among those with past COVID-19. We recommend that individuals with a poorly controlled DM and a recent episode of COVID-19 be screened for latent TB infection, and if positive, to consider LTBI treatment (italic font). **Bold numbers**, significant or borderline significant associations. Blue text in italics, clinical recommendations. Partially created with Biorender.

Our cross-sectional study revealed a novel relationship between a past symptomatic or asymptomatic COVID-19 episode on the odds of active TB. The prevalence of past COVID-19 was higher among newly diagnosed TB patients vs. non-TB controls, but only significant in hosts with DM, and strongest among those with poor glucose control. Likewise, we found that past COVID-19 magnified the strength of the known association between DM and TB. Namely, DM increased the odds of TB by 3-fold among non-COVID-19 participants, which is in line with pre-COVID-19 pandemic findings in our Mexico border community [13], and elsewhere [21]. However, the odds of TB increased to nearly 8-fold among participants with past-COVID-19. Together, these findings indicate an interaction between past COVID-19 and DM, magnifying the odds of TB when compared to COVID-19 or DM alone (Fig. 2).

The impact of the COVID-19 pandemic on higher TB incidence and mortality was predicted in the early stages of the COVID-19 pandemic, and partially attributed to programmatic shifts in resources from TB to COVID-19 [2, 22], but its biological impact is less clear [23]. In theory, past COVID-19 patients should be more vulnerable to TB due to reported viral induction of lymphopenia, functional T cell exhaustion, dysregulated innate and adaptive immune responses, and reduced secretion of IFN-γ in response to *Mtb* antigens [15, 16, 24]. Accordingly, some studies report cases of TB reactivation or primary TB attributed to a recent or concurrent COVID-19 episode [25]. However, our findings revealed a non-significant increase in the odds of TB in participants with past COVID-19 alone. There are several possible explanations for this low impact of COVID-19 on TB in our study: i) we only included past COVID-19 cases, and missed those with concurrent TB disease which is when highest immune compromise is most likely, and ii) we did not have information to stratify participants by COVID-19 disease severity, which seems critical for conferring TB risk [25].

DM has a known deleterious relationship with the risk and prognosis of COVID-19 or TB [17, 21]. The interaction between DM and COVID-19 on TB had been anticipated [3, 6, 26], and our observational study now support these predictions. The mechanism by which DM and COVID-19 synergize to magnify the risk of TB remains to be elucidated. It is possible that lymphopenia due to COVID-19 synergized with defective monocyte/macrophage effector functions in DM [27, 28] to compromise the cell-mediated immunity that is critical for *Mtb* containment [29].

Our findings have clinical implications. Individuals with a past COVID-19 episode and poor glucose control should be prioritized for evaluation of a latent TB infection in TB endemic regions, and if positive, offered latent TB infection treatment. This will be particularly important in recent contacts of a new TB case [30], or individuals who are not fully vaccinated against COVID-19, given that vaccines protect against the severity of this disease and dampen the COVID-19 related immune alterations that are likely to contribute to TB development risk [31].

We recognize study limitations. Our sample size is relatively small when considering the changing features of past COVID-19 individuals during the 18-month study period. These included the dwindling of the COVID-IgG titers as time elapsed between the COVID-19 and TB episodes, and the introduction of COVID-19 vaccines. It would have been ideal to stratify participants by these features, but our statistical power was insufficient. Our study design was cross-sectional with participants enrolled at the time of TB diagnosis, so past COVID-19 was based on indirect methods (self-report and serology) with possibilities for non-differential misclassifications. It would have been ideal to have clinical data to stratify COVID-19 severity or details on the vaccine manufacturer, doses, or timing. Our study may be prone to survival bias because we did not include those who succumbed to COVID-19 and/or TB. Despite these limitations, we were able to detect an anticipated interaction between DM and COVID-19 on the odds of TB.

## Conclusion

Looking forward, it may be uncertain how the COVID-19 pandemic will continue to impact the risk of TB given the acquisition of immunity to the virus through repeated infections in humans, plus the availability of second generation COVID-19 vaccines [32]. However, the emergence of new SARS-CoV-2 variants and low access to annual COVID-19 vaccines in TB endemic areas may retain the threat of higher TB risk, particularly among poorly controlled DM patients. Studies are warranted to confirm our findings with a larger sample size, in different study populations, at different times after a COVID-19 episode, and years after the emergence of the initial COVID-19 pandemic.

## Figures and Tables

**Table 1:** Relationship between variables documenting SARS-CoV-2 infection, COVID-19 disease and COVID-19 vaccination

	Anti-SARS-CoV-2 IgG index ≥1.4			Anti-SARS-CoV-2 IgG index ≥2.5		
	No	Yes	OR (95% CI)	No	Yes	OR (95% CI)
	n = 332	n = 113		n = 365	n = 80	
**Self-reported COVID-19**						
No	256 (82.8%)	53 (17.2%)	1.00	273 (88.3%)	36 (11.7%)	1.00
Yes	73 (57.0%)	55 (43.0%)	**3.6 (2.3–5.8)**	89 (69.5%)	39 (30.5%)	**3.3 (2.0–5.6)**
**COVID-19 Vaccination**						
No	174 (74.0%)	61 (26.0%)	1.00	184 (78.3%)	51 (21.7%)	1.00
Yes	67 (67.7%)	32 (32.3%)	1.4 (0.8–2.3)	79 (79.8%)	20 (20.2%)	0.9 (0.5–1.6)
**Self-reported COVID-19 × Vaccination**						
No disease, no vaccination	135 (83.9%)	26 (16.1%)	1.00	138 (85.7%)	23 (14.3%)	1.00
No disease, yes vaccination	47 (75.8%)	15 (24.2%)	1.7 (0.8–3.4)	56 (90.3%)	6 (9.8%)	0.6 (0.3–1.7)
Yes disease, no vaccination	36 (54.5%)	30 (45.5%)	**4.3 (2.3–8.2)**	43 (65.2%)	23 (34.9%)	**3.2 (1.6–6.3)**
Yes disease, yes vaccination	20 (54.0%)	17 (46.0%)	**4.4 (2.0–9.5)**	23 (62.2%)	14 (37.8%)	**3.7 (1.7–8.1)**

**Table 2:** Odds ratio of TB stratified by past COVID-19 and type 2 diabetes mellitus (DM) status 1

	No TB	TB	OR (95% CI)
**All participants**	**n = 373**	**n = 112**	
No DM	284 (85.3%)	49 (14.7%)	1.00
DM	89 (58.6%)	63 (41.4%)	**4.1 (2.6, 6.4)**
**Past COVID-19 negative**	**n = 242**	**n = 66**	
No DM	185 (83.7%)	36 (16.3%)	1.00
DM	57 (65.5%)	30 (34.5%)	**2.7 (1.5, 4.8)**
**Past COVID-19 positive**	**n = 131**	**n = 46**	
No DM	99 (88.4%)	13 (11.6%)	1.00
DM	32 (49.2%)	33 (50.8%)	**7.9 (3.7, 16.7)**

1 Past COVID-19 is based on reported COVID-19 or positive COVID-IgG at a 2.5 cut-off as described in the Results.

**Table 3:** Multivariable models for the adjusted odds ratio of TB disease in individuals with past COVID-19 1

Model	Population	Variables and interactions in final models ^2^	aOR of past COVID-19	p-value
**A**	All	All variables	1.55 (0.76–3.18)	0.228
**B**	All	Sex, BMI, DM	1.38 (0.78–2.46)	0.271
**C**	All	Sex, BMI, DM, past COVID-19 * DM		**0.057** ^3^
**D**	DM	Sex, BMI	**2.33 (0.97–5.57)**	**0.058**
**E**	HbA1c ≥ 7.0%	Sex, BMI	**2.99 (1.12–7.98)**	**0.029**
**F**	Hba1c ≥ 7.5%	Sex, BMI	**3.31 (1.19–9.22)**	**0.022**

1 Past COVID-19 is based on reported COVID-19 and COVID-IgG at a 2.5 cut-off as described in the Results

2 Model A includes all variables with p-value < 0.20 by bivariate analysis, namely: DM, sex, age distribution, BMI, marital status, COVID-19 vaccine, smoking, microvascular disease, triglyceride levels. Models B-F are reduced models with sex plus any variable remaining significant as listed for Model A, or the additional interaction term in Model C.

3 P-value for the interaction term.

Abbreviations: aOR, odds ratio of TB due to past COVID-19, after adjusting for the variables listed for each model; BMI, body-mass index; DM, type 2 diabetes mellitus; Bold numbers, significant or borderline significant values.

## Data Availability

The datasets used and/or analysed during the current study are available from the corresponding author on reasonable request.
